# Research Trends and Emerging Frontiers in Proteolysis Targeting Chimeras (PROTACs): A Bibliometric Analysis of 2630 Publications (2001–2025)

**DOI:** 10.3390/ph19070988

**Published:** 2026-06-25

**Authors:** Ganglin Su, Yihan Wang, Lin Yao

**Affiliations:** Department of Urology, Peking University First Hospital, Peking University, Beijing 100034, China

**Keywords:** Proteolysis Targeting Chimera, PROTACs, bibliometric analysis, targeted protein degradation, CiteSpace

## Abstract

**Background/Objectives:** Proteolysis Targeting Chimeras (PROTACs) are heterobifunctional small molecules that induce ubiquitin–proteasome–mediated degradation of target proteins and have matured from proof-of-concept chemistry to a clinically validated therapeutic modality, with the first Phase 3 readout reported in 2025. A systematic bibliometric analysis covering this pivotal-trial era, however, has been lacking. This study aimed to map the historical trajectory, current research front, and emerging frontiers of PROTAC research. **Methods:** We analyzed 2630 PROTAC-related publications indexed in the Web of Science Core Collection (WoSCC) from 2001 to 2025 using a combined toolkit of CiteSpace, HistCite, the Alluvial Generator, and R (ggplot2), covering co-occurrence networks, burst detection, keyword clustering, citation historiography, alluvial flow analysis, and reference co-citation timeline visualization. **Results:** China and the USA led global output, and the Chinese Academy of Sciences, China Pharmaceutical University, and Harvard University were the most productive institutions; the Journal of Medicinal Chemistry was the leading publishing venue, and Alessio Ciulli, Jian Jin, and Craig M. Crews anchored the author network. Keyword burst analysis showed that early research centred on E3 ubiquitin ligase recruitment and small-molecule PROTAC design, whereas the current hotspots, resolved through keyword clustering and co-citation timelines, included structural basis and ternary complex design, EGFR-directed degradation, oral bioavailability optimization, applications in multiple myeloma and Alzheimer’s disease, tumour-targeted delivery, and computational/AI-driven design. **Conclusions:** This study extends the bibliometric record of PROTACs across 2001–2025 and identifies oral bioavailability, E3 ligase repertoire expansion, and CNS-penetrant degrader design as the emerging frontiers likely to shape the next phase of the field.

## 1. Introduction

Proteolysis Targeting Chimeras (PROTACs) are heterobifunctional small molecules that tether a protein of interest (POI) to an E3 ubiquitin ligase, inducing polyubiquitination and proteasomal degradation of the target [1,2]. Unlike occupancy-driven inhibitors, PROTACs operate via an event-driven, catalytic mode whereby a single molecule iteratively recruits and eliminates multiple copies of the target, granting access to protein classes long regarded as “undruggable” [3]. The concept was established in 2001, when Sakamoto et al. [1] showed that a chimeric peptide could redirect an E3 ligase complex to degrade a chosen substrate. The field transitioned in 2015, when high-affinity small-molecule ligands for the VHL and CRBN E3 ligases enabled the design of fully drug-like PROTACs with nanomolar cellular potency [4,5]. Clinical translation began in 2019 with ARV-110 and ARV-471 entering Phase I trials, and in 2025 vepdegestrant (ARV-471) became the first PROTAC to deliver a statistically significant progression-free survival benefit in a Phase 3 trial (VERITAC-2), with more than 25 degrader candidates now in clinical development [6,7]. Alongside this accelerating translational trajectory, PROTAC-related publications have grown exponentially since 2015, with thematic boundaries continuing to expand; this growth underscores the need for a systematic bibliometric synthesis of the literature. Therapeutically, PROTACs have advanced farthest in oncology, with androgen-receptor degraders for prostate cancer and estrogen-receptor degraders for breast cancer being prime examples—the latter highlighted by the Phase 3 success of vepdegestrant. In parallel, a second wave of PROTACs is expanding into neurodegenerative disorders, including tau- and α-synuclein-directed degraders for Alzheimer’s and Parkinson’s disease. This trajectory underscores the broad therapeutic scope of the modality.

Bibliometric analysis is widely used to map the intellectual landscape of emerging therapeutic modalities. In the broader targeted protein degradation (TPD) field, the molecular glue literature has recently been mapped by two independent bibliometric studies [8,9], a pattern that reflects a community demand for structured knowledge maps of degrader technologies. PROTACs, the most clinically advanced TPD platform, have been the subject of only a single such analysis to our knowledge: Li et al. [10] analyzed 808 publications from 2001 to 2021 using CiteSpace 6.2.R4 and VOSviewer version 1.6.21. These studies rely on a narrow methodological toolkit, and the molecular glue analyses cannot substitute for a dedicated PROTAC synthesis: the two modalities differ substantially in E3-recruitment mechanism, design philosophy, and pace of clinical advancement. More importantly, the only PROTAC-focused bibliometric to date predates several pivotal developments that have reshaped the field over the past four years.

An updated, comprehensive, and methodologically broader bibliometric analysis of PROTACs is therefore needed. First, the coverage of Li et al. [10] ends before the field’s transition from proof-of-concept and early-phase trials to the pivotal-trial stage, missing both the first Phase 3 readouts and the expansion of the clinical pipeline to more than 25 molecules. Second, computational and AI-driven PROTAC design, together with the expansion of indications beyond oncology into neurodegenerative and infectious diseases, remain unresolved as distinct research clusters in prior bibliometric work. Third, earlier work largely relied on the CiteSpace–VOSviewer pairing, leaving the temporal flow of keyword evolution and the generational lineage of high-impact references incompletely mapped.

To address these gaps, we retrieved PROTAC-related publications indexed in the Web of Science Core Collection between 2001 and 2025 and integrated CiteSpace, HistCite, the Alluvial Generator, and R (ggplot2) for a multi-layered bibliometric analysis. The analysis focuses on (i) the historical features of the PROTAC literature, (ii) currently active research topics and their temporal evolution, and (iii) emerging trends likely to shape the next phase of PROTAC science. By combining an extended time window with a broader methodological toolkit, this study aims to offer researchers a systematic reference for mapping collaborative networks, tracking thematic evolution, and prioritizing research directions within the rapidly expanding PROTAC landscape. To orient the reader to the analyses that follow, we preview the emerging frontiers we ultimately identify: oral bioavailability optimization, expansion of the E3 ligase repertoire, CNS-penetrant degrader design, conditional and targeted delivery, and computational/AI-driven design. This preview allows the reader to track these themes as they emerge across the historical, thematic, and trend analyses.

## 2. Results

### 2.1. The Historical Features of the PROTACs Literature

#### 2.1.1. Distribution of Publications

**Figure 1:**
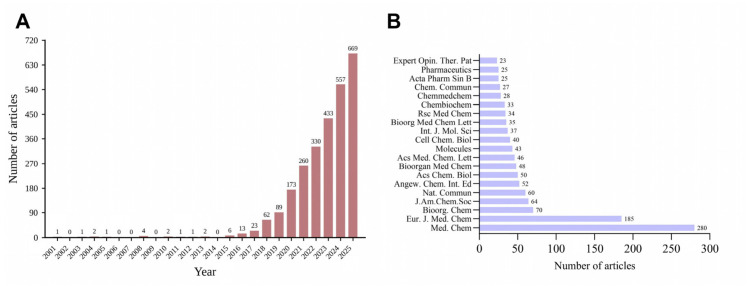
The historical features of publications on Proteolysis Targeting Chimera research. (**A**) The annual distribution of publications from 2001 to 2025. (**B**) The top 20 most fruitful journals ranked by publication count.

A total of 2630 publications related to Proteolysis Targeting Chimeras (PROTACs) were retrieved from the WoSCC database, comprising 1818 research articles and 812 reviews. The dataset involved 14,269 authors from 7679 institutions across 404 countries/regions, published in 516 journals spanning 82 subject categories (Table 1). The annual research output is illustrated in Figure 1A. Publication activity remained minimal from 2001 to 2015, with fewer than 10 papers per year. A marked acceleration began in 2016, when annual output first surpassed 10, and the growth rate intensified after 2019, with annual volumes climbing from 173 papers in 2019 to 669 in 2025. Among the publishing venues, the Journal of Medicinal Chemistry ranked first with 280 articles, followed by the European Journal of Medicinal Chemistry (185) and Bioorganic Chemistry (70). Figure 1B presents the top 20 most productive journals.

#### 2.1.2. Citation Structure of the PROTACs Research Field

**Figure 2:**
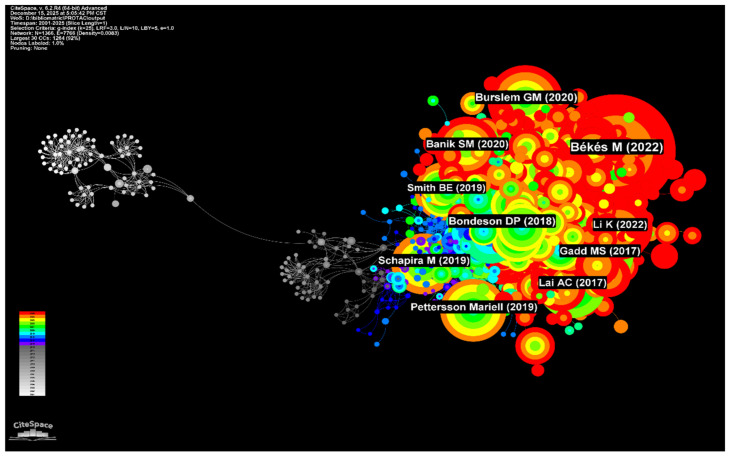
The citation co-occurrence network of Proteolysis Targeting Chimera research. Nodes represent cited references (size proportional to co-citation frequency), and the colour bar from left (cool colours) to right (warm colours) indicates the year from 2001 to 2025. Key landmark publications are labelled with author names and publication years [2,11,12,13,14,15]; the complete set of highly co-cited landmark references is provided in Table 2.

The co-citation network reveals the interconnections among publications in the PROTACs field over the past two decades (Figure 2). The network contains 1366 nodes and 7766 links. In the early period (2001–2015), grey-toned nodes form a densely connected foundation. During the middle period (2016–2020), blue-toned nodes were dispersed across the network. In the most recent period (2021–2025), nodes aggregated into tighter clusters, indicating the concentration and differentiation of research domains. Among these, Békés et al. [2], Burslem and Crews [11], and Bondeson et al. [12] occupied prominent positions with co-citation frequencies of 854, 350, and 291, respectively, followed by Gadd et al. [13] at 289, Lai and Crews [14] at 282, and Li and Crews [15] at 271.

The citation historiography was further mapped using HistCite Pro 2.1, and the landmark articles are listed in Table 2. The top three papers ranked by GCS were “Targeted intracellular protein degradation induced by a small molecule” (GCS = 2042), “Targeted protein degradation: expanding the toolbox” (GCS = 1838), and “Protacs: Chimeric molecules that target proteins to the Skp1-Cullin-F box complex for ubiquitination and degradation” (GCS = 1715).

#### 2.1.3. Scientific Cooperation

**Figure 3:**
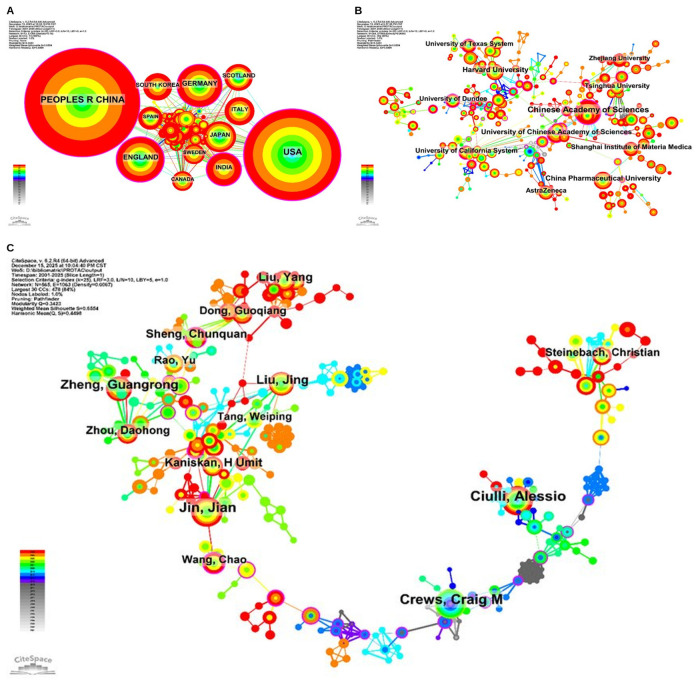
The scientific cooperation network in Proteolysis Targeting Chimera research. (**A**) Country cooperation network. Nodes represent countries (size proportional to publication volume), connecting lines indicate cooperative relationships, and the colour bar from left (cool colours) to right (warm colours) indicates the year from 2001 to 2025. (**B**) Institution cooperation network. Nodes represent institutions (size proportional to publication count), connecting lines indicate collaboration, and colours denote different time periods. (**C**) Author cooperation network. Nodes represent authors (size proportional to number of publications), links indicate co-authorship, and colours denote different collaborative clusters.

As shown in Figure 3, scientific collaborations were identified across three dimensions: country, institution, and author. The country cooperation network comprised 73 nodes and 368 links (Figure 3A). China occupied the largest node, followed by the USA, England, and Germany. At the institutional level, the cooperation network consisted of 364 nodes and 568 links (Figure 3B). The Chinese Academy of Sciences was the most productive institution, followed by China Pharmaceutical University, Harvard University, and the University of Chinese Academy of Sciences. The author collaboration network comprised 655 nodes and 1063 links (Figure 3C). Ciulli, Alessio, Jin, Jian, Crews, Craig M, and Zheng, Guangrong led in publication volume, and the dense linkages among them represent substantial co-authorship activity. The detailed co-occurrence frequencies for the top 10 countries, institutions, and authors are provided in Table S1.

### 2.2. Variation in the Most Active Topics

#### 2.2.1. Subject Category Burst

From 2001 to 2025, 64 of the 82 related subject categories experienced citation bursts. The category NANOSCIENCE and NANOTECHNOLOGY showed the strongest burst strength (5.5), with a burst period of 2024–2025. The temporal distribution of burst categories ranged from PEDIATRICS (2010) and CHEMISTRY, ORGANIC (2015–2016) in the early period to MATERIALS SCIENCE, MULTIDISCIPLINARY (2024–2025) and PHYSICS, APPLIED (2024–2025) in recent years. The detailed burst patterns for the top 50 subject categories are presented in Supplementary Figure S1, and the 20 categories with bursts persisting to 2025 are listed in Table S2.

#### 2.2.2. Keywords Burst

**Figure 4:**
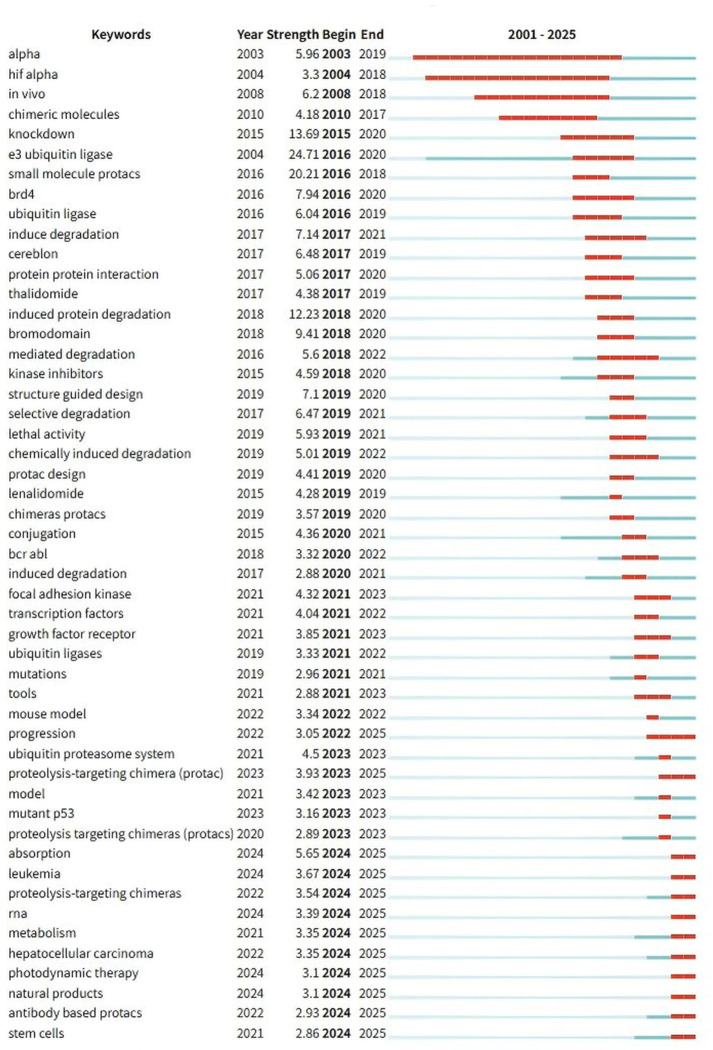
The top 50 keywords with the strongest citation bursts. Year indicates the first appearance of the keyword, Strength indicates the burst intensity, Begin and End indicate the start and end years of the burst period. The blue line represents the overall time interval from 2001 to 2025, and the red segments indicate the period during which the burst was active. Keywords are listed in the order in which their citation burst began, so that the figure can be read as a chronological account of shifting research foci across 2001–2025 rather than as an alphabetical index.

Keyword burst detection identified active research content throughout the study period. A total of 525 keywords exhibited citation bursts at different time points, and the top 50 with the strongest burst strength are displayed in Figure 4. The keyword “e3 ubiquitin ligase” had the highest burst strength (24.71), spanning 2016–2020, followed by “small molecule protacs” (Strength = 20.21, 2016–2018) and “knockdown” (Strength = 13.69, 2015–2020). Among all burst keywords, 20 remained active through 2025, including “absorption” (Strength = 5.65, 2024–2025), “proteolysis-targeting chimera (protac)” (Strength = 3.93, 2023–2025), “leukemia” (Strength = 3.67, 2024–2025), and “rna” (Strength = 3.39, 2024–2025). The complete list of keywords with bursts persisting to 2025 is available in Table S2.

#### 2.2.3. Reference Burst

A total of 1196 references with citation bursts were identified. The article with the strongest burst strength was “Phthalimide conjugation as a strategy for in vivo target protein degradation” by Winter et al. [5], which burst from 2016 to 2020 with a strength of 108.87. This study demonstrated that conjugating BET bromodomain antagonists with phthalimide moieties could hijack the cereblon E3 ubiquitin ligase complex, leading to highly selective ligand-dependent degradation of BET proteins both in vitro and in vivo. The second strongest burst belonged to “PROTAC targeted protein degraders: the past is prologue” by Békés et al. [2], bursting from 2024 to 2025 with a strength of 105.61. This review evaluated the first two decades of PROTAC development, assessed the current industrial landscape, and outlined future directions for targeted protein degradation. The third strongest burst was observed for “Catalytic in vivo protein knockdown by small-molecule PROTACs” by Bondeson et al. [4], with a strength of 104.91 from 2015 to 2020. This work reported two PROTACs capable of reducing protein levels by over 90% at nanomolar concentrations, with broad tissue distribution and target knockdown activity confirmed in tumour xenograft models. Among all burst references, 286 articles maintained active bursts through 2025, of which 14 were reviews and 6 were original articles in the top 20 by strength index. The detailed burst data are presented in Table S3.

### 2.3. Emerging Trends and New Developments

#### 2.3.1. The Temporal Variation in Keyword Clusters

**Figure 5:**
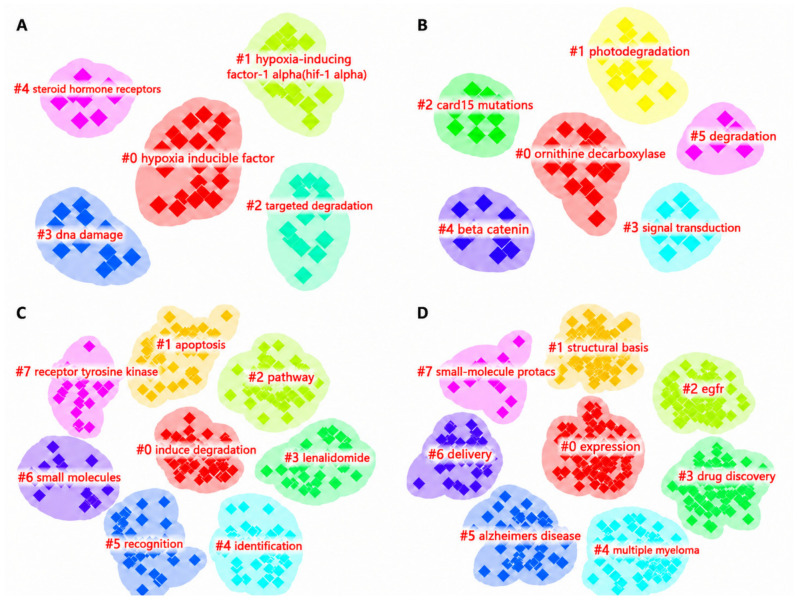
The keyword clustering snapshots in four periods. Each node represents a keyword, and colours denote different clusters labelled by the log-likelihood ratio algorithm. (**A**) 2001–2010, yielding 5 clusters including #0 hypoxia inducible factor, #1 hypoxia-inducing factor-1 alpha, #2 targeted degradation, #3 DNA damage, and #4 steroid hormone receptors. (**B**) 2011–2015, yielding 6 clusters including #0 ornithine decarboxylase, #1 photodegradation, #2 card15 mutations, #3 signal transduction, #4 beta catenin, and #5 degradation. (**C**) 2016–2020, yielding 8 clusters including #0 induce degradation, #1 apoptosis, #2 pathway, #3 lenalidomide, #4 identification, #5 recognition, #6 small molecules, and #7 receptor tyrosine kinase. (**D**) 2021–2025, yielding 9 clusters including #0 expression, #1 structural basis, #2 egfr, #3 drug discovery, #4 multiple myeloma, #5 alzheimers disease, #6 delivery, and #7 small molecule protacs.

Keywords with close internal associations can form distinct clusters based on their affinity, and the identification of these clusters helps to delineate the evolving research subfields in PROTACs. The 25-year study period was divided into four phases, and the keyword clustering snapshots for each phase are shown in Figure 5. In the first phase (2001–2010), 11 papers were included, yielding 5 clusters: #0 hypoxia-inducible factor, #1 hypoxia-inducing factor-1 alpha, #2 targeted degradation, #3 DNA damage, and #4 steroid hormone receptors (Figure 5A). The second phase (2011–2015) encompassed 10 papers and generated 6 clusters, including #0 ornithine decarboxylase, #1 photodegradation, and #2 card15 mutations (Figure 5B). In the third phase (2016–2020), the number of papers rose to 360, producing 8 clusters, such as #0 induce degradation, #1 apoptosis, and #2 pathway (Figure 5C). The fourth phase (2021–2025) incorporated 2249 papers and yielded 9 clusters: #0 expression, #1 structural basis, #2 egfr, #3 drug discovery, #4 multiple myeloma, #5 alzheimers disease, #6 delivery, and #7 small molecule protacs (Figure 5D). Compared with the preceding phases, classic topics such as DNA damage and targeted degradation persisted, while newly emerging clusters, namely #1 structural basis (80 articles), #2 egfr (72 articles), #3 drug discovery (70 articles), #4 multiple myeloma (46 articles), #5 alzheimers disease (46 articles), and #7 small molecule protacs (13 articles), attracted growing attention. The detailed cluster composition for the most recent phase is provided in Table S4.

#### 2.3.2. The Keyword Alluvial Flow Visualization

**Figure 6:**
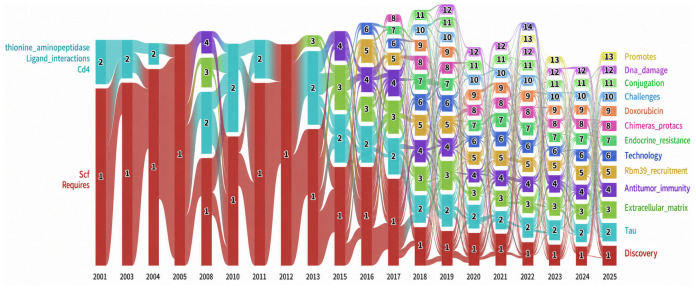
The keyword alluvial map from 2001 to 2025. Each vertical column corresponds to one annual time slice, with the horizontal axis running from 2001 on the left to 2025 on the right. Within each column, stacked blocks represent keyword modules, ordered from top to bottom by the number of constituent keywords (vertical axis). The integer label on each block denotes its rank within that year. Curved, coloured streams connect modules across adjacent years, tracing how keyword clusters merge, split, persist, or dissolve over time; stream width is proportional to the number of keywords carried forward between slices.

Figure 6 illustrates the keyword alluvial map from 2001 to 2025, in which linked keywords assemble into specific research modules that diverge or converge over time to form new modules. Some keywords maintained a strong presence throughout the entire study period, some emerged as new research trends, and others gradually faded. Module 1 in 2025 formed the largest tributary (highlighted in red). The most trafficked keywords for the top five modules each year are listed in Table S5. The detailed keyword composition of the top 6 modules in 2024 is further examined in the following section.

#### 2.3.3. Detailed Composition of Emerging Keyword Modules

**Figure 7:**
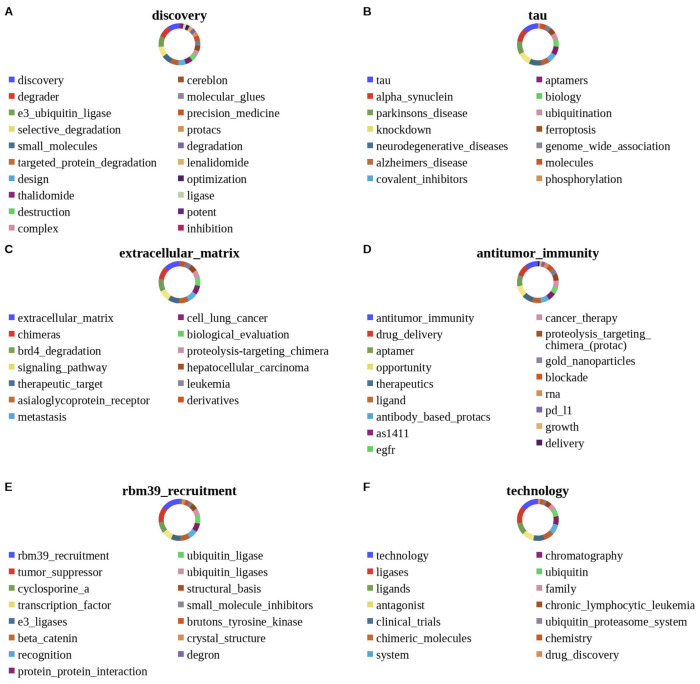
The keywords of the top 6 modules in 2024 were derived from the alluvial flow analysis. Each donut chart displays the constituent keywords of a module, where the arc length of each segment is proportional to the relative weight of the keyword within that module. (**A**) Module 1, named “discovery”, gathers 20 keywords such as degrader, e3_ubiquitin_ligase, and selective_degradation. (**B**) Module 2, named “tau”, gathers 14 keywords such as alpha_synuclein, parkinsons_disease, and neurodegenerative_diseases. (**C**) Module 3, named “extracellular_matrix”, gathers 13 keywords such as chimeras, brd4_degradation, and signaling_pathway. (**D**) Module 4, named “antitumor_immunity”, gathers 17 keywords such as drug_delivery, aptamer, and antibody_based_protacs. (**E**) Module 5, named “rbm39_recruitment”, gathers 15 keywords such as tumor_suppressor, cyclosporine_a, and protein_protein_interaction. (**F**) Module 6, named “technology”, gathers 14 keywords such as ligases, ligands, and ubiquitin_proteasome_system. In every panel, the arc length of each coloured segment is proportional to that keyword’s relative weight (frequency) within the module. The module name displayed above each donut chart corresponds to the module’s highest-weighted keyword, as determined by the log-likelihood ratio labelling algorithm.

To characterize the emerging research directions, the keyword composition of the top 6 modules in 2024 was examined (Figure 7). Module 1 was designated “discovery,” gathering 20 keywords such as degrader, e3_ubiquitin_ligase, and selective_degradation (Figure 7A). Module 2, named “tau,” contained 14 keywords including alpha_synuclein, parkinsons_disease, and neurodegenerative_diseases (Figure 7B). Module 3, labelled “extracellular_matrix,” comprised 13 keywords such as chimeras, brd4_degradation, and signaling_pathway (Figure 7C). Module 4, termed “antitumor_immunity,” assembled 17 keywords including drug_delivery, aptamer, and antibody_based_protacs (Figure 7D). Module 5 was identified as “rbm39_recruitment,” encompassing 15 keywords such as tumor_suppressor, cyclosporine_a, and protein_protein_interaction (Figure 7E). Module 6, named “technology,” gathered 14 keywords including ligases, ligands, and ubiquitin_proteasome_system (Figure 7F).

#### 2.3.4. The Timeline Visualization of References

**Figure 8:**
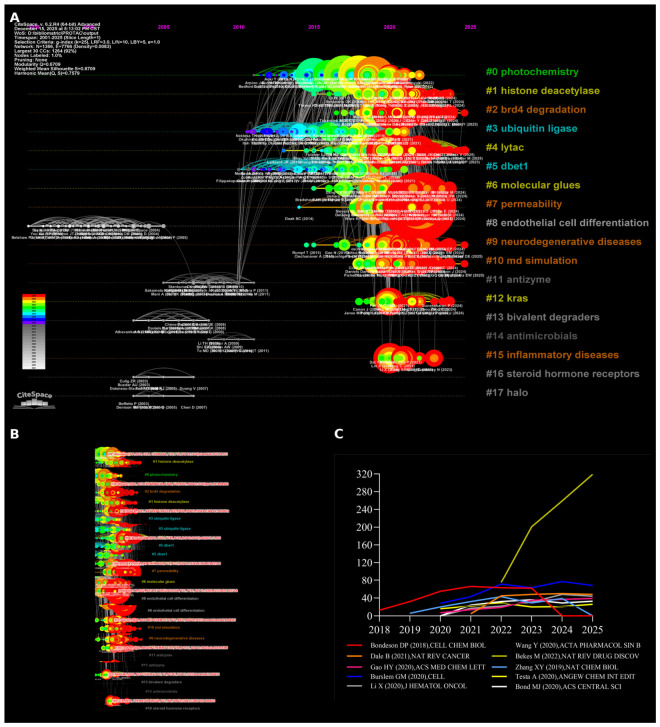
The reference clusters map. (**A**) The citation timeline visualization. Clusters are arranged top-down by size, and the horizontal axis represents the timeline from 2001 to 2025. Nodes represent cited references (size proportional to citation frequency); larger nodes with red rings indicate key publications with citation bursts, and the colour bar from left (cool colours) to right (warm colours) indicates the publication year. (**B**) The burst citations in representative clusters. Each panel highlights a landmark publication (labelled with co-citation frequency, author, year, journal, and DOI) within its corresponding cluster, showing the citation context and connections to neighbouring references. These highlighted landmark publications are cited in the main text and listed in Table 2 [2,11,12,13,14,16,17,18,19,20,21]. (**C**) Citation frequency distribution of the burst citations from 2018 to 2025, where each line represents a key publication and the vertical axis indicates annual cited frequency. Across all panels, node size is proportional to citation frequency, and node colour follows the year colour bar, with cool colours indicating earlier years and warm colours indicating more recent ones. Red rings mark references with citation bursts. Lines between nodes denote co-citation links.

A timeline visualization based on reference co-citation analysis was constructed to distinguish emerging, classic, and relatively outdated topics in PROTACs research. The timeline map consisted of 18 clusters arranged top-down by size (Figure 8A). Clusters #3 ubiquitin ligase and #5 dbet1 represented classic topics that maintained extensive connections to other clusters throughout the timeline. Clusters #8 endothelial cell differentiation, #11 antizyme, #13 bivalent degraders, #14 antimicrobials, #16 steroid hormone receptors, and #17 halo were relatively outdated topics, characterized by sparse inter-cluster connections and no sustained development on their own timelines. Clusters #0 photochemistry, #1 histone deacetylase, #2 brd4 degradation, #4 lytac, #6 molecular glues, #7 permeability, #9 neurodegenerative diseases, #10 md simulation, #12 kras, and #15 inflammatory diseases were identified as emerging topics, as they remained active from their first appearance through the present.

Several landmark publications with citation bursts anchored these emerging subfields (Figure 8B). Bondeson et al. [12], belonging to cluster #0 with a co-citation frequency of 291, revealed that PROTAC selectivity is governed by the stability of the ternary complex formed between E3 ligase and target protein, rather than binary binding affinity alone. Wang et al. [22], in cluster #1 with a co-citation frequency of 129, reviewed recent advances in PROTAC design, including the first ternary co-crystal structure of BRD4–PROTAC–VHL and the entry of ARV-110 into clinical trials. Dale et al. [16], in cluster #2 with a co-citation frequency of 196, assessed degrader candidates targeting cancer-relevant proteins, with emphasis on exploiting underexplored E3 ligases for tissue-specific degradation. Burslem and Crews [11], in cluster #4 with a co-citation frequency of 350, introduced PROTACs as versatile tools for biological discovery and compared PROTAC-mediated protein regulation with RNA interference and genome editing. Zhang et al. [17], in cluster #6 with a co-citation frequency of 169, identified DCAF16 as a target for electrophilic PROTACs that promote nucleus-restricted protein degradation through covalent engagement. Békés et al. [2], in cluster #7 with a co-citation frequency of 854, assessed the first twenty years of PROTAC development and outlined key directions for expanding targeted protein degradation beyond oncology. Gao et al. [18], in cluster #8 with a co-citation frequency of 151, reviewed opportunities and challenges of PROTAC technology, covering early clinical candidates such as ARV-110, strategies for expanding the E3 ligase toolbox, and key design considerations including cell permeability and ternary complex stability. Testa et al. [19], in cluster #10 with a co-citation frequency of 134, demonstrated that macrocyclization of PROTACs can enhance degradation potency and selectivity through conformational constraint of the ternary complex. Bond et al. [20], in cluster #12 with a co-citation frequency of 153, reported LC-2, the first PROTAC capable of degrading endogenous KRAS(G12C), achieving sustained target knockdown and MAPK pathway suppression. Li and Song [21], in cluster #15 with a co-citation frequency of 162, reviewed PROTAC-mediated degradation of oncogenic proteins with a focus on hematological malignancies. The citation frequency distribution of these ten articles from 2018 to 2025 is shown in Figure 8C. The detailed information on all emerging clusters is available in Table S6.

## 3. Discussion

### 3.1. General Information

The trajectory of PROTAC publication activity aligns closely with the field’s translational milestones (Figure 2). Output was confined to a small circle of laboratories for nearly fifteen years after the original small-molecule PROTAC proposal by Sakamoto et al. [1]. The inflection beginning in 2016 coincides with the disclosure of drug-like VHL ligands capable of disrupting VHL/HIF-1α binding in cells [23] and the demonstration that phthalimide-based cereblon recruitment could achieve potent in vivo BET-protein degradation [5]. A second inflection after 2019, when annual output expanded roughly fourfold within six years, mirrors the clinical entry of ARV-110 (bavdegalutamide) and ARV-471 (vepdegestrant) [6]. This curve is plausibly set to steepen further: vepdegestrant achieved a statistically significant progression-free survival benefit over fulvestrant in the Phase 3 VERITAC-2 trial, and its New Drug Application is now under FDA review [7].

The 2001–2021 record of Li et al. [10] terminated on the ascending limb of this curve, capturing the chemistry-to-clinic transition but missing the pivotal-trial phase now visible in our data. Our dataset therefore extends the bibliometric record across the first regulatory-stage inflection in the modality’s history.

The geographical pattern observed for PROTACs research differs from the “country-of-origin” asymmetry typical of ethnopharmacology. PROTACs originated in the Crews laboratory at Yale, from which both the conceptual framework and the first-generation degraders emerged, and American dominance in the earliest literature therefore reflects technological parentage rather than geography. The later ascendance of China to the leading position by publication volume (Figure 3A) aligns with a broader reorientation of the Chinese biopharmaceutical sector toward innovation-driven small-molecule drug discovery, in which targeted protein degradation has been explicitly identified as a priority modality [24]. The substantial co-authorship traffic between Chinese and American institutions (Figure 3A,B) is therefore better interpreted as collaborative consolidation than as parallel competition. At the disciplinary level, the dominance of Journal of Medicinal Chemistry and European Journal of Medicinal Chemistry as top publishing venues (Figure 1B) reinforces that PROTAC research remains, at its core, a medicinal-chemistry problem, one centred on linker engineering, E3-ligand optimization, and physicochemical rescue of molecules that routinely lie beyond the rule-of-five space [25]. Within the author network, Ciulli and Crews anchor two complementary contributions: VHL-centred structural and cooperativity work from Dundee [13] and the founding lineage of degrader medicinal chemistry. The dense inter-group edges of the collaboration map (Figure 3C) indicate that the field, although numerically large (14,269 authors), remains socially connected through a relatively small backbone of core laboratories.

Taken together, these patterns indicate that the bibliometric trajectory functions as a leading indicator of translational maturation. The quantitative shifts our analysis detects—the post-2019 acceleration in output, the recent burst of “absorption,” and the consolidation of disease-specific clusters such as multiple myeloma and Alzheimer’s disease—track the field’s movement from proof-of-concept chemistry toward pivotal-trial-stage therapeutics, mirroring the clinical advance of degraders such as vepdegestrant from first-in-human dosing to a positive Phase 3 readout. Read in this way, the publication record is not merely a description of scholarly activity, but a quantitative reflection of the modality’s clinical coming-of-age, and the emerging clusters identified below can be interpreted as early signals of the directions most likely to reach the clinic next.

We now examine the keyword-level structure that resolves the research front into distinct thematic clusters.

### 3.2. Emerging Research Hotspots

#### 3.2.1. Structural Basis and Rational Design of PROTACs

The most consequential conceptual shift our analysis surfaces in the recent literature is the turn toward the structural and thermodynamic basis of degrader action—a theme that emerges as one of the dominant keyword clusters of the most recent phase (Figure 5D, cluster #1, 80 articles). This turn is reinforced by the persistent activity of timeline cluster #0 (photochemistry and VHL/CRBN structural work), which has remained active since its first appearance and harbours several of the most highly co-cited references in the field (Figure 8A,B).

Although structure-centred work was already present in the 2001–2021 landscape [10], the present record shows a clear expansion in both cluster size and thematic breadth, consistent with the conceptual pivot over the past four years from warhead affinity toward ternary complex geometry as the primary design variable.

This pivot rests on the demonstration that cooperativity between the E3 ligase and the target protein, rather than binary binding affinity alone, determines degradation potency and selectivity. The BRD4BD2/VHL/MZ1 ternary structure illustrated how degrader-induced protein–protein contacts generate a favourable cooperative surface [13]; in this sense, the active species is a supramolecular assembly of degrader, target protein, and E3 ligase, whose collective stability—rather than the affinity of either binary interaction alone—dictates productive ubiquitin transfer. Selectivity among closely related paralogs has since been shown to depend primarily on ternary complex stability rather than on warhead specificity [12]. Two design strategies emerged directly from this insight. Linker rigidification, exemplified by ternary-complex-crystal-structure-guided replacement of a flexible linker with a conformationally rigid moiety in a second-generation VHL-based BCL-xL/BCL-2 dual degrader, improved both potency and degradation kinetics [26]; macrocyclization, first applied to MZ1 to generate macroPROTAC-1 [19] and recently extended to “head-to-tail” scaffolds that largely dismiss the hook effect [27], constrains the bioactive conformation and reduces entropic penalties on ternary complex formation. A complementary direction addresses the narrow E3 ligase repertoire. Although more than 600 E3 ligases are encoded in the human genome, most published degraders still recruit CRBN or VHL, a concentration also visible as Module 1 (“discovery”) in our alluvial map (Figure 6). Recent chemoproteomic campaigns have progressively enlarged the ligandable toolbox to include DCAF16, DCAF11, RNF114, KEAP1, FEM1B, and, most recently, DCAF1, with the last harnessed to degrade cancer-relevant targets such as WDR5 and offering a means to mitigate the loss of degradation efficacy that arises when CRBN or VHL is downregulated or mutated [28,29]. The disclosure of intramolecular bivalent glues, in which a single small molecule engages two sites on a target to induce an E3-recruiting conformation [30], further blurs the operational boundary between PROTACs and molecular glues.

These structural and chemoproteomic advances are now feeding back into computational workflows, with physics-based ternary modelling approaching near-crystallographic accuracy for several VHL and CRBN systems [31]; the computational side of this feedback loop is examined in Section 3.2.7.

#### 3.2.2. Overcoming Drug Resistance via Targeted Degradation

A second major axis of recent activity is the use of targeted degradation to overcome drug resistance, a problem for which event-driven degraders are mechanistically well suited; this theme forms the second-largest keyword cluster of the most recent phase (Figure 5D, cluster #2, 72 articles), led thematically by “EGFR.” The co-citation timeline further situates this cluster at the intersection of the catalytic proof-of-concept literature [4] and the mechanistic framing provided by Burslem and Crews [11], the latter occupying a central position in the field’s co-citation network (Figure 8B).

Although EGFR and KRAS were already discernible as emerging targets in the 2001–2021 landscape [10], their consolidation into a dedicated resistance-oriented cluster is a feature of the past four years, paralleling the clinical arrival of PROTAC degraders explicitly designed for on-target mutations that disable conventional inhibitors.

The mechanistic rationale is that event-driven degradation, in which a single molecule triggers ubiquitination of multiple target copies before being released, is, in principle, insensitive to target upregulation and to most active-site mutations. This advantage is most clinically resonant for EGFR: first-line osimertinib controls EGFR-mutant non-small cell lung cancer effectively, yet the C797S on-target mutation abolishes covalent TKI binding and leaves patients without a clear next-line option. PROTAC degraders of the Del19/T790M/C797S and L858R/T790M/C797S triple mutants have now been reported with nanomolar DC50 values and in vivo activity in osimertinib-resistant patient-derived xenograft models, including the orally bioavailable HJM-561 and fourth-generation reversible EGFR binders repurposed as warheads [32,33]. KRAS has followed a similar arc on a shorter timescale: the degradation of endogenous KRAS(G12C) by LC-2 [20] was followed within four years by a pan-KRAS degrader active against 13 of 17 clinically relevant mutants [34] and by the selective G12D degrader ASP3082, whose VHL ternary complex has been crystallographically resolved [35]. Several KRAS degraders have now entered Phase 1 trials.

A candid reading of the mechanism also acknowledges that PROTACs are not themselves immune to resistance: genomic alterations in CRBN and other core E3 machinery have been reported in cell lines chronically exposed to BET degraders, which partly motivates the E3 ligase diversification discussed in Section 3.2.1.

#### 3.2.3. Medicinal Chemistry Optimization and Oral Bioavailability

The physicochemical properties of PROTACs—molecular size, polarity, hydrogen-bonding capacity, and conformational flexibility—are the principal determinants of their cellular behaviour, governing membrane permeation, intracellular availability, and ultimately oral exposure. Within this dimension, oral bioavailability has shifted from being regarded as the defining liability of the modality to a tractable medicinal-chemistry problem, a transition our data capture quantitatively: the keyword “absorption” registers one of the strongest active bursts in the PROTAC literature (burst strength 5.65, 2024–2025; Table S2), coinciding with the growth of the “drug discovery” cluster to 70 articles in the most recent phase (Figure 5D, cluster #3).

Whereas Li et al. [10] identified oral-bioavailability concerns largely at the level of qualitative narrative, the present record shows this theme resolving into a quantitatively dominant burst, reflecting the transition from an acknowledged problem to an actively solved medicinal-chemistry exercise.

PROTACs, with molecular weights routinely in the 800–1100 Da range and high counts of hydrogen-bond donors, acceptors, and rotatable bonds, populate the “beyond rule-of-five” (bRo5) chemical space where Lipinski-based prediction breaks down [25]. The dominant conceptual framework to emerge is chameleonicity: the capacity of a molecule to collapse into a compact, hydrogen-bond-shielded conformation in nonpolar environments and to unfold in aqueous settings [36]. Intramolecular hydrogen bonding that masks polar surface area during membrane permeation has been invoked to rationalize the oral bioavailability of bavdegalutamide and vepdegestrant, both of which populate 3D polar-surface-area and radius-of-gyration distributions consistent with chameleonic behaviour [37]. Industrial analyses of internal PROTAC collections have refined bRo5-specific guidelines, identifying the Caco-2 efflux ratio as a practical screening criterion [38]. Complementary work has refined composite physicochemical descriptors that capture polarity masking and has systematically mapped the chemical space of orally bioavailable candidates [39]. The medicinal-chemistry campaign that produced ARV-766 from the ARV-110 scaffold illustrates how linker re-engineering and stereochemical refinement at the cereblon-binding moiety can convert a marginal-exposure degrader into one with clinical-grade pharmacokinetics, and the oral VHL-based SMARCA2 degrader ACBI2 extends the same logic to the more hydrophilic VHL ligase [40].

Oral bioavailability, once viewed as the defining liability of the modality, is now a tractable problem with articulated design rules, a transition that the burst profile captures quantitatively.

#### 3.2.4. PROTACs in Oncology: Hematological Malignancies and Beyond

“Multiple myeloma” stands out with 46 articles in the most recent disease-specific cluster (Figure 5D, cluster #4). The co-citation timeline shows persistent co-occurrence between BRD4-degradation work (cluster #2) and inflammatory-disease work (cluster #15), both of which harbour landmark reviews that disproportionately address hematological targets (Figure 8B).

Oncology was already the dominant therapeutic area in the 2001–2021 landscape [10]; what has changed is the distribution within oncology. The multiple-myeloma signal, relatively subtle in the earlier mapping, has consolidated into a defined cluster, and clinical-stage degraders have multiplied beyond the two AR/ER exemplars that anchored the earlier record.

The prominence of myeloma is a historical consequence of the entanglement between its pharmacopeia and the CRBN E3 ligase. Immunomodulatory drugs such as lenalidomide and pomalidomide were established myeloma therapies long before their mechanism of CRBN-mediated neo-substrate degradation was understood, and repurposing the same CRBN-binding pharmacophore as the E3-recruiting warhead of heterobifunctional degraders has anchored a large share of PROTAC medicinal chemistry to this ligase [16]. Clinical translation now extends well beyond myeloma itself. In breast cancer, vepdegestrant’s positive Phase 3 outcome and NDA filing represent the closest approach any degrader has made to regulatory approval (Section 3.1). In B-cell malignancies, the BTK degrader NX-2127 operationalizes a distinct design principle: simultaneous degradation of BTK and the transcription factors IKZF1/IKZF3, coupling a target-directed effect with immunomodulatory activity reminiscent of lenalidomide itself [41]. Phase 1 data have demonstrated sustained BTK degradation exceeding 80% across mutant genotypes, including kinase-impaired C481S variants resistant to both covalent and noncovalent BTK inhibitors, with clinical responses in a majority of heavily pretreated patients [42]. BGB-16673 and NX-5948 are concurrently advancing through Phase 1/2 studies.

Across the field, more than 25 PROTAC or molecular-glue degraders are now in human trials, most directed against oncology targets [43]. Whether PROTAC-inhibitor combinations can synergize without overlapping toxicities will largely determine how deeply degradation-based pharmacology embeds itself in routine oncology practice.

#### 3.2.5. PROTACs for Neurodegenerative Diseases

Neurodegenerative targets converge across three independent indicators: the “Alzheimer’s disease” keyword cluster (Figure 5D, cluster #5, 46 articles), the “tau” alluvial module (Figure 7B, Module 2, 14 keywords), and the “neurodegenerative diseases” timeline cluster that has remained active to the present (Figure 8A, cluster #9).

This is a genuinely new feature of the landscape. In the 2001–2021 mapping of Li et al. [10], neurodegenerative disease did not emerge as a distinct keyword cluster; its resolution as an independent, multi-indicator theme in the current record marks one of the clearest examples of thematic diversification since that baseline.

Tau and α-synuclein are two intrinsically disordered proteins whose pathological aggregation drives Alzheimer’s disease and Parkinson’s disease, respectively [44], and both now anchor much of the CNS degrader literature. Silva et al. [45] provided early evidence that small-molecule PROTACs could selectively degrade aberrant tau in frontotemporal dementia patient-derived neurons without affecting wild-type tau in healthy controls, using a CRBN-recruiting degrader built around the PET tracer scaffold T807. Optimized tau PROTACs subsequently reduced both total and hyperphosphorylated tau in transgenic mouse models with measurable improvements in cognitive performance [46,47]. Small-molecule and peptide-based PROTACs targeting α-synuclein have subsequently been reported, and proof-of-concept degradation has recently been extended to mutant huntingtin. CNS-targeted PROTACs face constraints distinct from those encountered in oncology. Blood–brain barrier penetration imposes hard physicochemical limits on polar surface area, hydrogen-bond donor count, and efflux susceptibility, compounding the bRo5 difficulties discussed in Section 3.2.3 [48]. The catalytic advantage of PROTAC degradation is especially attractive in this context because the low CNS drug concentrations imposed by the BBB can still achieve therapeutic target engagement if each molecule triggers multiple rounds of degradation.

The Phase 1 entry of ARV-102, an LRRK2-targeting PROTAC for Parkinson’s disease and progressive supranuclear palsy, marks the first clinical test of degradation logic in a neurodegenerative indication. Whether this approach can succeed where decades of small-molecule and antibody-based tau therapies have fallen short remains open, but the convergence of structural biology, patient-derived models, and clinical-stage chemistry places the field closer to a definitive answer than at any previous point.

#### 3.2.6. Delivery Strategies and Precision Activation

“Delivery” is one of the emerging clusters of the most recent phase (Figure 5D, cluster #6), and the defining keywords of alluvial Module 4, namely “drug_delivery,” “aptamer,” and “antibody_based_protacs” (Figure 7D), resolve the same theme at a different bibliometric layer, indicating convergence across independent analytical methods.

Delivery was essentially absent as an organized theme in the 2001–2021 record [10]. Its coordinated emergence across both keyword clustering and alluvial decomposition is therefore a second genuinely new feature of the current landscape, alongside neurodegenerative disease.

The driver is recognition that on-target, off-tissue toxicity limits systemically administered degraders: because E3 ligases are expressed in both diseased and healthy cells, a potent PROTAC reaching non-target tissues will degrade its substrate indiscriminately. This concern first emerged with BET degraders, for which systemic BRD4 depletion in rodents produced skin and metabolic toxicity. Two broad classes of solutions have emerged. The first attaches PROTACs to tumour-homing macromolecules such as antibodies or aptamers, which restrict cellular entry to receptor-expressing cells. Degrader–antibody conjugates (DACs) borrow the linker–payload architecture of ADCs to deliver cleavable PROTACs after receptor-mediated endocytosis, with ORM-5029 now in Phase 1 evaluation. Aptamer–PROTAC conjugates offer a complementary format with lower immunogenicity and simpler manufacturing; an AS1411 aptamer–BET PROTAC conjugate achieved selective BRD4 degradation in nucleolin-positive breast cancer cells while sparing normal tissue in xenograft models [49]. The second class employs prodrug strategies in which a caged PROTAC is activated only upon exposure to a tumour-specific stimulus such as light, hypoxia, reactive oxygen species, or ionizing radiation, confining degradation to a spatially or microenvironmentally defined volume [50]. These conditional approaches intersect with nanoparticle-based delivery: sequentially responsive nano-PROTACs have been engineered to exploit successive tumour-microenvironment and intracellular cues for precise intracellular delivery and enhanced degradation efficacy [51]. More broadly, encapsulation and nanoformulation—packaging degraders within polymeric micelles, stimulus-responsive nanoparticles, or carrier conjugates—directly mitigate the poor aqueous solubility, limited membrane permeability, and on-tissue/off-target toxicity that constrain free PROTACs. The prominence of this theme in our most recent period (Figure 5D, cluster #6; Figure 7D) indicates that formulation-based strategies have become an organized research front rather than isolated proofs of concept. Beyond restricting which cells a degrader enters, these delivery and activation strategies also provide a means of controlling its subcellular fate: stimulus-triggered uncaging and carrier release can bias degraders toward the intracellular compartments where their target proteins and the recruited E3 machinery reside, an emerging design consideration that parallels the permeability-focused work captured by reference cluster #7 (Figure 8A).

Although most platforms remain preclinical, their rapid proliferation suggests degrader delivery is entering the optimization cycle that ADCs underwent a decade earlier, with combinatorial possibilities expanding as new E3 warheads and targeting ligands become available.

#### 3.2.7. Computational Approaches: MD Simulation and AI-Driven Design

Timeline cluster #10, labelled “md simulation,” appears late in the record but remains active through the present (Figure 8A). Its emergence indicates the growing role of physics-based and data-driven computation in degrader design.

Computational PROTAC design was not resolved as a distinct cluster in the 2001–2021 landscape [10]. Its appearance as an independent, still-active cluster in the current record is the third new structural feature we identify, completing, together with neurodegenerative disease (Section 3.2.5) and delivery (Section 3.2.6), the three thematic expansions that most clearly distinguish the 2022–2025 period from the earlier baseline.

Molecular dynamics simulations have moved from a supporting role in rationalizing known ternary complexes to a prospective one: all-atom MD trajectories are now used to rank linker candidates by predicted cooperativity and to estimate the conformational ensemble that determines whether a given degrader will adopt a productive geometry for ubiquitin transfer. Recent docking-plus-refinement protocols achieve near-native ternary complex predictions for multiple VHL and CRBN systems [31]. In parallel, machine-learning models trained on curated degrader databases are beginning to address the combinatorial explosion inherent in PROTAC design, where warhead, linker, and E3 ligand can each be varied independently. Graph-neural-network architectures and generative chemistry pipelines have been applied to predict DC50 values, cell permeability, and degradation selectivity from molecular graphs, although predictive accuracy remains inferior to that achieved for conventional small molecules because training sets are smaller and ternary-complex pharmacology introduces variables absent from classical QSAR [10].

Our bibliometric data indicate that the PROTAC field now exceeds 2600 publications, and as the number of experimentally characterized degraders continues to grow, data-driven methods are likely to improve rapidly. Their integration with physics-based ternary modelling may ultimately enable closed-loop automated degrader design, an endpoint anticipated by the “md simulation” cluster’s trajectory but not yet realized.

### 3.3. Limitations

Several limitations of this bibliometric study should be acknowledged. First, all publications were retrieved exclusively from the WoSCC database; articles indexed only in Scopus, PubMed, or regional databases were therefore not captured, which may underrepresent contributions published in non-English languages or in journals outside the WoSCC coverage. We acknowledge that multi-database designs—most commonly combining Web of Science with Scopus—have become the more robust standard in contemporary bibliometric practice, as they improve coverage completeness and reduce single-source selection bias. Our reliance on the Web of Science Core Collection (WoSCC) alone was guided primarily by methodological compatibility. The co-citation and citation-historiography analyses central to this study—performed using CiteSpace and HistCite—depend on the structured “Cited References” records that WoSCC provides. Harmonizing cited-reference data across WoSCC and Scopus without introducing duplication or matching artifacts was beyond what could be reliably achieved in this study. Consequently, the absolute publication and citation figures reported in this study should therefore be interpreted as representing a consistent sample within WoSCC rather than as an exhaustive census of the field. A future multi-database analysis incorporating Scopus would provide valuable cross-validation of the trends identified in this study. Second, the retrieval was restricted to peer-reviewed research articles and reviews, excluding preprints, conference abstracts, and patent literature. These sources carry disproportionate weight in a fast-moving translational field where industrial disclosures often precede formal publication. Third, the 2025 data were collected before the end of the calendar year and thus reflect an incomplete annual output; the true publication volume for 2025 is likely to be higher than reported. Fourth, CiteSpace-based clustering relies on co-citation and co-occurrence algorithms whose outputs are sensitive to parameter settings and time-slicing intervals, and the cluster labels are generated automatically from citing articles rather than being assigned by domain experts, which may occasionally produce labels that do not precisely capture the intellectual content of a cluster. Fifth, our keyword-based clustering depends on author-supplied keywords, and the PROTAC literature exhibits substantial terminological heterogeneity: “PROTAC,” “PROTACs,” “proteolysis-targeting chimera,” “protac degrader,” and related variants have all been used as author keywords for conceptually identical entities. Although we applied a keyword-cleaning protocol modelled on Li et al. [10] to consolidate synonyms and abbreviations, residual dilution of cluster signals cannot be ruled out, particularly for adjacent concepts such as “molecular glue degrader” and “bifunctional degrader,” whose boundary with PROTAC proper has blurred since the intramolecular bivalent glue framework was introduced (Hsia et al. 2024 [30]; Section 3.2.1). Despite these constraints, the dataset of 2630 publications spanning twenty-four years, analyzed through complementary bibliometric tools, provides a systematic and reproducible overview of the PROTACs research landscape and offers a reliable foundation for identifying the field’s historical trajectory, collaborative structure, and emerging frontiers.

## 4. Materials and Methods

### 4.1. Data Collection and Statistics

Publications were retrieved from the Web of Science Core Collection (WoSCC). The search strategy was: TS=(“Proteolysis Targeting Chimera”) OR TS=(PROTACs) OR TS=(“PROTAC Proteolysis Targeting Chimera”) OR TS=(PhosphoPROTACs) OR TS=(“VHL-Based PROTACs”) OR TS=(“IAP-Based PROTACs”) OR TS=(“Cereblon-Based Small-Molecule Compounds”) OR TS=(“MDM2 Protein-Based PROTACs”), with the time span set from 2001 to 2025. The initial search returned 2976 records, which were filtered by document type to retain only “Articles” and “Reviews” (2640 records), and further restricted to English-language publications, yielding a final dataset of 2630 records. All retrieval was performed on 25 December 2025 to avoid bias caused by daily database updates. The records were exported as plain text files in “Full Record and Cited References” format and are hereafter referred to as DATA. Country, institution, journal, author, and document-type counts were tabulated in Microsoft Excel 2019 (Microsoft Corporation, Redmond, WA, USA) for descriptive statistics.

### 4.2. Tools for Bibliometric Analysis

#### 4.2.1. CiteSpace

CiteSpace (version 6.2.R4) was the primary tool for visualization and network analysis in this study [52].

##### Co-Occurrence Networks

Co-occurrence refers to the simultaneous appearance of multiple authors, institutions, or countries/regions within the same publication [53]. CiteSpace renders these relationships as co-occurrence networks that reveal collaborative patterns across three dimensions: authorship, institution, and country. In the network visualization, node colour encodes the year in which the corresponding entity first appeared, edge colour encodes the year the co-occurrence link was first formed, and each node is represented by concentric rings, with ring thickness proportional to the frequency of co-occurrence in a given year. A red ring marks a citation burst in that year, and a purple ring denotes high betweenness centrality.

##### Burst Detection

Based on Kleinberg’s algorithm [54], Chen and colleagues formalized citation bursts as indicators of active research topics [52]. A citation burst indicates that a subject category, keyword, or reference has attracted a sharp increase in attention over a defined time window. CiteSpace provides burst detection for subject categories, keywords, and references.

##### Cluster Analysis

CiteSpace implements three clustering algorithms operating on titles, abstracts, and keywords, grouping publications into conceptual clusters with distinct thematic characteristics [55]. The cluster map depicts concept cluster composition across time periods; the timeline view traces the emergence, persistence, and decline of each cluster together with its connections to adjacent clusters.

##### Parameter Settings

DATA was imported into CiteSpace with “Time Slicing” set to 2001–2025 and “1 year per slice.” The term sources were “Title,” “Abstract,” “Author Keywords (DE),” and “Keywords Plus.” Node types were selected according to the analytical objective, with all other parameters left at the default. Collaboration networks for countries/regions, institutions, and authors were generated automatically and manually adjusted for readability. For keyword clustering, the node type was set to “Keywords,” and the time span was divided into four slices: 2001–2010, 2011–2015, 2016–2020, and 2021–2025. The first interval was defined as a longer ten-year window because publication output during 2001–2010 was minimal, with fewer than ten papers per year (Figure 1A). Consolidating these sparse early years into a single phase yields an interpretable clustering snapshot. For reference co-citation analysis, the “Timeline View” was selected under the “Layout” tab to render the co-citation timeline. Burst detection maps for keywords, subject categories, and references were generated via the “Burstness” tab.

#### 4.2.2. HistCite

HistCite Pro 2.1 was used to map the citation historiography of the PROTACs field. The software scores publications by Local Citation Score (LCS), the citation count within the imported dataset, and Global Citation Score (GCS), the citation count across the entire WoSCC. DATA (2630 records) was imported into HistCite Pro 2.1 with “Limit” set to 30 and all other parameters left at default. The “Make Graph” function was used to render the citation network and identify influential publications.

#### 4.2.3. Alluvial Generator

Alluvial flow diagrams were used to trace temporal patterns in the evolving keyword network [56]. Keyword co-occurrence networks for each time slice were first generated in CiteSpace, then exported and loaded into the Alluvial Generator (Mapequation, Umeå University; available at http://www.mapequation.org/apps/AlluvialGenerator.html, accessed on 25 December 2025). Each keyword was treated as a node and clustered within each time slice, with each cluster treated as a module. Nodes split and merged across time slices to form new modules, and the longest-lived nodes were highlighted by colouring the flows they trace.

#### 4.2.4. R

The donut charts depicting the keyword composition of emerging modules (Figure 7) were plotted in R (version 4.2.2) using the geom_bar function from the ggplot2 package (version 3.4.4).

### 4.3. Use of Generative AI

The authors used a generative artificial intelligence tool (Claude, Anthropic, Claude Opus 4.8) for language editing and formatting assistance during manuscript preparation. The tool was used to improve grammar, academic expression, readability, and parts of the document formatting. All scientific content, data analysis, interpretation, and conclusions were generated and verified by the authors, who take full responsibility for the integrity and accuracy of the work.

## 5. Conclusions

This bibliometric analysis mapped the research landscape of PROTACs from 2001 to 2025, resolving both the collaborative architecture of the field and its thematic evolution. The data describe a field in rapid expansion, with priorities shifting from foundational mechanistic work toward disease-oriented applications and clinical translation. Although PROTACs have reached clinical proof-of-concept and have yielded their first Phase 3 readout in 2025, substantial work remains before degraders become a routine therapeutic modality.

Five directions will shape the next phase of translation. Oral bioavailability remains constrained by the high molecular weight and poor membrane permeability of degraders; macrocyclization and chameleonic design are the principal strategies now available to improve pharmacokinetic profiles without sacrificing degradation potency. Expanding the E3 ubiquitin ligase repertoire beyond CRBN and VHL, toward ligases such as DCAF16, RNF114, and KEAP1, is needed to achieve tissue-selective and disease-specific degradation. For neurodegenerative indications, CNS-penetrant PROTACs capable of crossing the blood–brain barrier require coordinated computational modelling and experimental optimization of physicochemical properties. Conditional activation strategies, including photoswitchable prodrugs and antibody- or aptamer-PROTAC conjugates, may mitigate on-target, off-tissue toxicity and widen the therapeutic window. Finally, coupling molecular dynamics simulations with machine-learning frameworks could shift PROTAC design from empirical screening toward rational, structure-guided discovery. Collectively, these directions define the trajectory from catalytic proof-of-concept toward clinically deployable degraders.

## Figures and Tables

**Table 1:** Basic information on the distribution of the publications.

Categories	Publication	Articles	Review	Authors	Institutions	Journals	Subject Categories
Amount	2630	1818	812	14,269	7679	516	82

**Table 2:** The information on the top 30 literature sorted by LCS. LCS: the total local citation score; GCS: the total global citation score; NO: the number of the literature in the database imported into HistCite Pro 2.1.

NO	Article Information	Journal	LCS	GCS
1	Protacs: Chimeric molecules that target proteins to the Skp1-Cullin-F box complex for ubiquitination and degradation	P NATL ACAD SCI USA	947	1715
705	PROTAC targeted protein degraders: the past is prologue	NAT REV DRUG DISCOV	854	946
20	Catalytic in vivo protein knockdown by small-molecule PROTACs	NAT CHEM BIOL	593	758
41	Structural basis of PROTAC cooperative recognition for selective protein degradation	NAT CHEM BIOL	539	698
36	Induced protein degradation: an emerging drug discovery paradigm	NAT REV DRUG DISCOV	512	1565
17	Hijacking the E3 Ubiquitin Ligase Cereblon to Efficiently Target BRD4	CHEM BIOL	473	645
18	Selective Small Molecule-Induced Degradation of the BET Bromodomain Protein BRD4	ACS CHEM BIOL	461	503
62	Lessons in PROTAC Design from Selective Degradation with a Promiscuous Warhead	CELL CHEM BIOL	426	566
27	PROTAC-induced BET protein degradation as a therapy for castration-resistant prostate cancer	P NATL ACAD SCI USA	377	499
254	Proteolysis-Targeting Chimeras as Therapeutics and Tools for Biological Discovery	CELL	350	582
24	Small-Molecule PROTACS: New Approaches to Protein Degradation	ANGEW CHEM INT EDIT	325	772
61	The Advantages of Targeted Protein Degradation Over Inhibition: An RTK Case Study	CELL CHEM BIOL	322	654
197	Targeted protein degradation: expanding the toolbox	NAT REV DRUG DISCOV	314	1838
8	Targeted intracellular protein degradation induced by a small molecule: En route to chemical proteomics	BIOORG MED CHEM LETT	314	2042
23	Modular PROTAC Design for the Degradation of Oncogenic BCR-ABL	ANGEW CHEM INT EDIT	313	989
778	PROTACs: past, present and future	CHEM SOC REV	271	868
93	Plasticity in binding confers selectivity in ligand-induced protein degradation	NAT CHEM BIOL	269	1102
309	Lysosome-targeting chimeras for degradation of extracellular proteins	NATURE	258	903
196	A selective BCL-XL PROTAC degrader achieves safe and potent antitumor activity	NAT MED	247	804
47	Targeted protein degradation by PROTACs	PHARMACOL THERAPEUT	233	653
3	Chemical genetic control of protein levels: Selective in vivo targeted degradation	J AM CHEM SOC	231	657
163	BAF complex vulnerabilities in cancer demonstrated via structure-based PROTAC design	NAT CHEM BIOL	224	750
151	Targeted protein degradation: elements of PROTAC design	CURR OPIN CHEM BIOL	216	522
65	Protac-Induced Protein Degradation in Drug Discovery: Breaking the Rules or Just Making New Ones?	J MED CHEM	215	502
98	Delineating the role of cooperativity in the design of potent PROTACs for BTK	P NATL ACAD SCI USA	211	475
34	Protein Degradation by In-Cell Self-Assembly of Proteolysis Targeting Chimeras	ACS CENTRAL SCI	208	668
166	Proteolysis targeting chimeras (PROTACs) in ‘beyond rule-of-five’ chemical space: Recent progress and future challenges	BIOORG MED CHEM LETT	207	496
19	HaloPROTACS: Use of Small Molecule PROTACs to Induce Degradation of Halo Tag Fusion Proteins	ACS CHEM BIOL	207	496
95	Targeting the C481S Ibrutinib-Resistance Mutation in Bruton’s Tyrosine Kinase Using PROTAC-Mediated Degradation	BIOCHEMISTRY-US	204	443
132	Discovery of ARD-69 as a Highly Potent Proteolysis Targeting Chimera (PROTAC) Degrader of Androgen Receptor (AR) for the Treatment of Prostate Cancer	J MED CHEM	198	783

## Data Availability

All bibliographic data analyzed in this study are publicly accessible through the Web of Science Core Collection. The processed datasets and analysis files generated in this study are available from the corresponding author on reasonable request.

## Supplementary Materials

The following supporting information can be downloaded at: https://www.mdpi.com/article/10.3390/ph19070988/s1. Figure S1: The top 50 subject categories with the strongest citation bursts (Year indicates the first appearance of the category, Strength indicates the burst intensity, Begin and End indicate the start and end years of the burst period; the blue line represents the overall time interval from 2001 to 2025, and the red segments indicate the period during which the burst was active); Table S1: The top 10 countries, institutions, and authors ranked by frequency of co-occurrence (CoF: co-occurrence frequency; Year: year of first collaboration appearance); Table S2: The top 20 subject categories and keywords with citation bursts persisting from beginning to 2025 (Begin: the burst beginning year; End: the burst ending year; Strength: the burst strength index; Year: the first appearance time; Entity: the term); Table S3: The references with citation bursts at different periods; Table S4: Summary of keyword clusters for the most recent stage (2021–2025) (Size: the number of articles in each cluster; Silhouette: the average contour value of clustering; LLR: log-likelihood ratio); Table S5: The most trafficked keyword for the top five modules each year; Table S6: Summary of emerging topics from the citation timeline visualization (Size: the number of articles in each cluster; Silhouette: the average contour value of clustering; LLR: log-likelihood ratio).

## Abbreviations

The following abbreviations are used in this manuscript:

AI	Artificial Intelligence
ADC	Antibody–Drug Conjugate
BBB	Blood–Brain Barrier
BET	Bromodomain and Extra-Terminal motif
bRo5	beyond Rule of Five
CNS	Central Nervous System
CRBN	Cereblon
DAC	Degrader–Antibody Conjugate
EGFR	Epidermal Growth Factor Receptor
GCS	Global Citation Score
LCS	Local Citation Score
MD	Molecular Dynamics
PROTAC	Proteolysis Targeting Chimera
TPD	Targeted Protein Degradation
VHL	von Hippel–Lindau
WoSCC	Web of Science Core Collection
