# Supplementary material for: Research Trends and Emerging Frontiers in Proteolysis Targeting Chimeras (PROTACs): A Bibliometric Analysis of 2630 Publications (2001–2025)
Source: Pharmaceuticals (Basel). 2026 Jun 25;19(7):988. doi: 10.3390/ph19070988 (PMC13415214; doi:10.3390/ph19070988)
Supplement: Supplementary file 1 [file pharmaceuticals-19-00988-s001.zip › Supplementary Materials.pdf]

Supplementary materials

Table S1. The top 10 countries, institutions and authors for frequency of co-occurrence.

CoF	Year	Country	CoF	Year	Institution	CoF	Year	Author
1122	2013	CHINA	179	2018	Chinese Academy of Sciences	49	2015	Ciulli, Alessio
824	2001	USA	99	2018	China Pharmaceutical University	46	2020	Jin, Jian
205	2015	ENGLAND	93	2017	Harvard University	46	2008	Crews, Craig M
178	2015	GERMANY	88	2019	University of Chinese Academy of Sciences	33	2020	Zheng, Guangrong
106	2012	INDIA	65	2001	University of California System	30	2020	Liu, Jing
104	2015	JAPAN	65	2021	Shanghai Institute of Materia Medica	29	2016	Demizu, Yosuke
88	2016	ITALY	64	2018	AstraZeneca	28	2016	Naito, Mikihiko
75	2018	SOUTH KOREA	59	2013	Tsinghua University	23	2020	Liu, Yang
74	2015	SCOTLAND	59	2015	University of Dundee	21	2022	Sheng, Chunquan
50	2018	CANADA	58	2018	University of Texas System	21	2021	Kaniskan, H Umit

CoF: Co-occurrence frequency. Year:Year of first collaboration appearance

Table S2. The top 20 subject categories and keywords burst with a burst period from beginning to 2025.

Subject category bursts					Keywords bursts				
Begin	End	Strength	Year	Entity	Begin	End	Strength	Year	Entity
2024	2025	5.5	2020	NANOSCIENCE & NANOTECHNOLOGY	2024	2025	5.65	2024	absorption
2024	2025	2.43	2021	MATERIALS SCIENCE, MULTIDISCIPLINARY	2023	2025	3.93	2023	proteolysis-targeting chimera (protac)
2024	2025	1.93	2004	CHEMISTRY, APPLIED	2024	2025	3.67	2024	leukemia
2024	2025	1.79	2023	POLYMER SCIENCE	2024	2025	3.39	2024	rna
2024	2025	1.61	2021	PHYSICS, APPLIED	2024	2025	3.35	2022	hepatocellular carcinoma
2024	2025	1.37	2023	PHYSICS, CONDENSED MATTER	2024	2025	3.35	2021	metabolism
2024	2025	0.88	2020	MATERIALS SCIENCE, BIOMATERIALS	2024	2025	3.1	2024	natural products
2024	2025	0.62	2024	GASTROENTEROLOGY & HEPATOLOGY	2024	2025	3.1	2024	photodynamic therapy
2023	2025	0.61	2023	PLANT SCIENCES	2024	2025	3.05	2022	progression
2022	2025	0.55	2022	COMPUTER SCIENCE, ARTIFICIAL INTELLIGENCE	2022	2025	2.93	2022	antibody based protacs
2024	2025	0.47	2024	SOCIAL SCIENCES, INTERDISCIPLINARY	2024	2025	2.86	2021	stem cells
2024	2025	0.47	2024	INTEGRATIVE & COMPLEMENTARY MEDICINE	2024	2025	2.84	2017	cancer cells
2024	2025	0.47	2024	COMPUTER SCIENCE, SOFTWARE	2024	2025	2.82	2024	pancreatic cancer

				ENGINEERING					
2024	2025	0.47	2024	CELL & TISSUE ENGINEERING	2024	2025	2.76	2022	methylation
2024	2025	0.47	2024	AGRONOMY	2024	2025	2.75	2023	permeability
2024	2025	0.47	2024	AGRICULTURE, MULTIDISCIPLINARY	2024	2025	2.7	2019	protac
2024	2025	0.41	2024	ENTOMOLOGY	2023	2025	2.68	2023	ligands
2024	2025	0.41	2024	CARDIAC & CARDIOVASCULAR SYSTEMS	2023	2025	2.62	2023	target protein degradation
2024	2025	0.34	2022	PHYSIOLOGY	2023	2025	2.54	2024	kinase inhibitor
-	-	-	-	-	2024	2025	2.51	2021	protac degrader

Bigin: the burst’ beginning year, End: the burst’ ending year, Strength: the burst’ strength index, Year: the first appearance time, Entity: the term.

Table S3

References	Year	Strength	Begin	End	2000 - 2025
Bondeson DP, 2015, NAT CHEM BIOL, V11, P611, DOI 10.1038/NCHEMBIO.1858, DOI	2015	104.91	**2015**	2020	
Buckley DL, 2015, ACS CHEM BIOL, V10, P1831, DOI 10.1021/acschembio.5b00442, DOI	2015	40.45	**2015**	2020	
Winter GE, 2015, SCIENCE, V348, P1376, DOI 10.1126/science.aab1433, DOI	2015	108.87	**2016**	2020	
Lu J, 2015, CHEM BIOL, V22, P755, DOI 10.1016/j.chembiol.2015.05.009, DOI	2015	95.37	**2016**	2020	
Zengerle M, 2015, ACS CHEM BIOL, V10, P1770, DOI 10.1021/acschembio.5b00216, DOI	2015	79.82	**2016**	2020	
Lai AC, 2016, ANGEW CHEM INT EDIT, V55, P807, DOI 10.1002/anie.201507634, DOI	2016	71.95	**2016**	2021	
Toure M, 2016, ANGEW CHEM INT EDIT, V55, P1966, DOI 10.1002/ange.201507978, DOI	2016	64.68	**2016**	2021	
Fischer ES, 2014, NATURE, V512,	2014	33.19	**2016**	2019	

P49, DOI 10.1038/nature13527, DOI				
Galdeano C, 2014, J MED CHEM, V57, P8657, DOI 10.1021/jm5011258, DOI	2014	31.98	**2016**	2019
Lu G, 2014, SCIENCE, V343, P305, DOI 10.1126/science.1244917, DOI	2014	27.15	**2016**	2019
Krönke J, 2014, SCIENCE, V343, P301, DOI 10.1126/science.1244851, DOI	2014	25.94	**2016**	2019
Raina K, 2016, P NATL ACAD SCI USA, V113, P7124, DOI 10.1073/pnas.1521738113, DOI	2016	75.58	**2017**	2021
Lai AC, 2017, NAT REV DRUG DISCOV, V16, P101, DOI 10.1038/nrd.2016.211, DOI	2017	65.16	**2017**	2022
Lebraud H, 2016, ACS CENTRAL SCI, V2, P927, DOI 10.1021/acscentsci.6b00280, DOI	2016	30.36	**2017**	2021
Matyskiela ME, 2016, NATURE, V535, P252, DOI 10.1038/nature18611, DOI	2016	27.52	**2017**	2021
Ottis P, 2017, ACS CHEM BIOL, V12, P892, DOI 10.1021/acschembio.6b01068, DOI	2017	23.76	**2017**	2021

Gadd MS, 2017, NAT CHEM BIOL, V13, P514, DOI 10.1038/nchembio.2329, DOI	2017	68.1	**2018**	2022
Bondeson DP, 2018, CELL CHEM BIOL, V25, P78, DOI 10.1016/j.chembiol.2017.09.010, DOI	2018	33.86	**2018**	2021
Zhou B, 2018, J MED CHEM, V61, P462, DOI 10.1021/acs.jmedchem.6b01816, DOI	2018	31.77	**2018**	2020
Neklesa TK, 2017, PHARMACOL THERAPEUT, V174, P138, DOI 10.1016/j.pharmthera.2017.02.027, DOI	2017	28.46	**2018**	2022
Robb CM, 2017, CHEM COMMUN, V53, P7577, DOI 10.1039/c7cc03879h, DOI	2017	27.26	**2018**	2021
Zorba A, 2018, P NATL ACAD SCI USA, V115, PE7285, DOI 10.1073/pnas.1803662115, DOI	2018	26.32	**2019**	2021
Sun YH, 2018, CELL RES, V28, P779, DOI 10.1038/s41422-018-0055-1, DOI	2018	25.34	**2019**	2022
Békés M, 2022, NAT REV DRUG DISCOV, V21, P181, DOI 10.1038/s41573-021-00371-6, DOI	2022	105.61	**2024**	2025
Chirnomas D, 2023, NAT REV CLIN	2023	55.39	**2024**	2025

ONCOL, V20, P265, DOI 10.1038/s41571-023-00736-3, DOI					
Li K, 2022, CHEM SOC REV, V51, P5214, DOI 10.1039/d2cs00193d, DOI	2022	52.91	**2024**	2025	
Liu Z, 2022, MOL BIOMED, V3, P0, DOI 10.1186/s43556-022-00112-0, DOI	2022	37.68	**2024**	2025	
Zhao L, 2022, SIGNAL TRANSDUCT TAR, V7, P0, DOI 10.1038/s41392-022-00966-4, DOI	2022	32.74	**2024**	2025	
Guenette RG, 2022, CHEM SOC REV, V51, P5740, DOI 10.1039/d2cs00200k, DOI	2022	24.8	**2024**	2025	
Chen Y, 2022, CHEM SOC REV, V51, P5330, DOI 10.1039/d1cs00762a, DOI	2022	23.77	**2024**	2025	

Table S5. Summary of keyword clusters for the most recent stage(2021-2025).

**ClusterID**	**Size**	**Silhouette**	**Average Year**	**Label (LLR)**	**Representative keywords**
0	83	0.553	2022	expression	expression; cancer; gene; therapy; targets | protein; degradation; discovery; inhibitor; family
1	80	0.722	2021	structural basis	targeted protein degradation; proteolysis-targeting chimeras; linker flexibility; oncogenic proteins; transcription factor | protein degradation; molecular glue; rational design; md simulation; biodegradation efficiency
2	72	0.654	2022	egfr	drug resistance; molecular glue; hematologic malignancies; mantle cell; transcription factor |

					targeted therapy; molecular mechanisms; prostate cancer; tumor microenvironment; androgen receptor
3	70	0.601	2022	drug discovery	drug discovery; absorption; permeability; solubility; rule | targeted protein degradation; targeting chimera; multiple myeloma; transcription factor; partition coefficients
4	46	0.679	2022	multiple myeloma	molecular glue; natural product; hydrophobic tagging; retinoblastoma protein; bifunctional molecule | breast cancer; endocrine therapy; estrogen receptors; hormone-dependent tumors; hormonal signaling pathways
5	46	0.672	2022	alzheimers disease	targeted protein degradation; parkinsons disease; lewy bodies; dbpp; toolbox | alzheimers disease; tau hyperphosphorylation; kirsten rat sarcoma; kras inhibitors; viral oncogene homolog
6	35	0.751	2023	delivery	cancer therapy; stat3 degraders; small molecule inhibitors; engineering methods; lysosomal degradation | targeted protein degradation; proteolysis-targeting chimera; ubiquitin-proteasome system; nano-based tpd technology; stat3 degraders
7	13	0.852	2021	small molecule protacs	protein degradation; fragment-based ligand discovery; targeted cancer therapy; small molecule; targeted therapies | small molecule; e3 ubiquitin; aryl hydrocarbon; anaplastic lymphoma; focal adhesion

Size: the number of articles in each cluster; Silhouette: the average contour value of clustering, it is generally believed that the clustering category with S > 0.5 is reasonable, and S > 0.7 means that the clustering is convincing; LLR: Log-likelihood ratio.

Table S6. The most trafficked keyword for the top five modules each year.

Year	2000	2001	2002	2003	2004	2005	2006	2007	2008	2009
Total modules	-	2	-	6	7	6	-	-	11	-
module 1	-	scf	-	proteolysis	estrogen_receptor_(er)	chemical_genetics	-	-	estrogen_receptor	-
module 2	-	methionine_aminopeptidase	-	estrogen_receptor	androgen_receptor	-	-	-	degradation	-
module 3	-	-	-	-	-	-	-	-	chemical_inducers_of_dimerization	-
module 4	-	-	-	-	-	-	-	-	chemically_induced_dimerization	-
module 5	-	-	-	-	-	-	-		-	-
**Year**	2010	2011	2012	2013	2014	2015	2016	2017	2018	2019
Total modules	15	16	13	14	-					
module	antiestrogen	ubiquitinat	saccharomyces	nerve_growth_f	-	estrogen_recept	design	induce_degra	estrogen_r	selective_degradati

1		ion	_cerevisiae	actor		or_alpha		dation	eceptor	on
module 2	methionine_aminopeptidase	degradation	-	mechanisms	-	conjugation	protein_degradation	induced_degradation	e3_ubiquitin_ligase	translation
module 3	-	-	-	molecules	-	targeted_degradation	biochemical_techniques_and_resources	cereblon	acquired_radioresistance	e3_ubiquitin_ligase
module 4	-	-	-	-	-	b_cell_lymphoma	axonal_transport	androgen_receptor_gene	mediated_degradation	protein-protein_interactions
module 5	-	-	-	-	-	-	knockdown	antitumor_activity	protein_protein_interaction	complex
**Year**	2020	2021	2022	2023	2024	-2025	-	-	-	-
Total modules	15	16	13	14	14	-33	-	-	-	-
module 1	acquired_resistance	btk_inhibitors	selective_degradation	autophagy	cancer_hallmarks	discovery	-	-	-	-
module 2	selective_degradation	selective_degradation	induce_degradation	elacestrant_rad1901	post-translational_modification	tau	-	-	-	-
module 3	degrader	alpha_synuclein	methylation	proteasome	tyrosine_kinase_in	extracellular_matrix	-	-	-	-

					hibitor					
module 4	immunomodulatory_drugs	therapeutic_target	combination_therapy	highly_potent	lenalidomide	antitumor_immunity	-	-	-	-
module 5	chemical_biology	tumor_suppressor	mediated_degradation	biological_evaluation	molecular_mechanisms	rbm39_recruitment	-	-	-	-

Table S7. Summary of emerging topics.

ClusterID	Size	Silhouette	Average Year	Label (LLR)	Representative keywords
0	154	0.892	2017	photochemistry	protein degradation; ubiquitin-proteasome system; e3 ubiquitin ligase; heterobifunctional molecule; target protein | targeted protein degradation; targeting chimera; tumor microenvironment; tumor immunotherapy; computational strategies
1	153	0.699	2020	histone deacetylase	targeted protein degradation; targeting chimera; small molecule inhibitors; tumor microenvironment; tumor immunotherapy | protein degradation; e3 ligase; ubiquitin proteasome system; shp2 degradation; thalidomide-based heterobifunctional molecules
2	122	0.921	2021	brd4 degradation	targeted protein degradation; precision medicine; chimeric degraders; extracellular protein clearance; lysosome-targeting chimeras | protein degradation; e3 ligase; ubiquitin proteasome system; solid tumors; castration resistance
4	106	0.855	2020	lytac	targeted protein degradation; precision medicine; lysosome-targeting chimeras; chimeric degraders; extracellular protein clearance | protein degradation; ubiquitin-proteasome system; e3 ubiquitin ligase; heterobifunctional molecule; target protein
6	103	0.843	2021	molecular glues	targeted protein degradation; neurodegenerative disease; ubiquitin code; immune disorder; p3 properties | protein degradation; molecular glue; small molecule; bivalent degraders; lysosomal degradation
7	77	0.886	2021	permeability	targeted protein degradation; drug discovery; precision cancer therapy; off-tissue toxicity; smart protacs | protein degradation; ubiquitin proteasome system; e3 ligase; neurodegenerative disease; proteolytic targeting chimeras
9	58	0.873	2021	neurodegenerative diseases	targeted protein degradation; high-throughput imaging; transcription factor; protein knockdown; small molecule | protein degradation; drug delivery system; proteolytic targeting

					chimeras; drug discovery; tumorigenesis
10	48	0.931	2021	md simulation	targeted protein degradation; assay development; dynamic combinatorial chemistry; protac; drug resistance | protein degradation; ubiquitin proteasome system; e3 ligase; ubiquitin; degradation
12	20	0.98	2021	kras	targeted protein degradation; hematological malignancies; kras inhibitor; kras vaccine; krasg12c inhibitors | lung cancer; ubiquitin-proteasome system; precision oncology; kras inhibitor; kras vaccine
15	9	0.997	2021	inflammatory diseases	targeted protein degradation; ubiquitin ligase; protacs technology; cancer treatment; small molecule | protein degradation; ubiquitin proteasome system; e3 ligase; hsp90 chaperone; therapy-resistant nsclc
