# Supplementary figures and images for: Research Trends and Emerging Frontiers in Proteolysis Targeting Chimeras (PROTACs): A Bibliometric Analysis of 2630 Publications (2001–2025)
Source: Pharmaceuticals (Basel). 2026 Jun 25;19(7):988. doi: 10.3390/ph19070988 (PMC13415214; doi:10.3390/ph19070988)

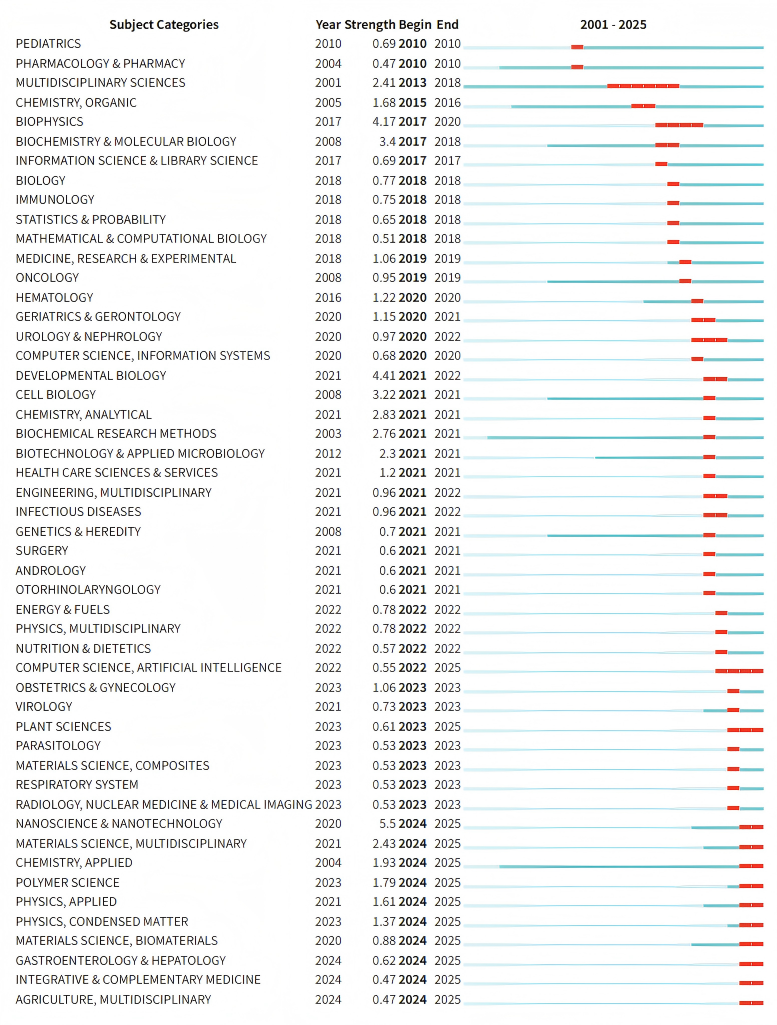

Supplement: Supplementary file 1 [file pharmaceuticals-19-00988-s001.zip › Supplementary Fig. S1(1).png]
